# Frequency of ubiquitous connectivity and associated factors among Mexican adolescents

**DOI:** 10.1186/s12911-019-0922-9

**Published:** 2019-11-15

**Authors:** Arturo Aguilar-Ye, Hortensia Reyes-Morales, Lourdes Campero, Nicéforo Garnelo-Bibiano

**Affiliations:** 1 Escuela de Salud Pública de Médico, Instituto Nacional de Salud Pública, Av. Universidad 655, CP 62100 Cuernavaca, Morelos Mexico; 2 Centro de Investigación en Sistemas de Salud, Instituto Nacional de Salud Pública, Av. Universidad 655, CP 62100 Cuernavaca, Morelos Mexico; 3 Centro de Investigación en Salud Poblacional, Instituto Nacional de Salud Pública. Cuernavaca, Av. Universidad 655, CP 62100 Cuernavaca, Morelos Mexico; 4 Centro de Investigación en Evaluación y Encuestas, Instituto Nacional de Salud Pública, Av. Universidad 655, CP 62100 Cuernavaca, Morelos Mexico

## Abstract

**Background:**

There is limited information in Mexico - a middle-income country and a digital adopter with an important demographic bonus - regarding the potential use of technology and connectivity in health promotion among adolescent population.

Therefore, the objective of this study was to determine the proportion of adolescents connected ubiquitously; and to identify its associated factors for the further development of mobile health interventions.

**Methods:**

An online survey of adolescents from state of Morelos, Mexico, was conducted in 2016. Explored individual socio-educational and school technological infrastructure characteristics and habits of use of mobile technologies. A logistic regression model was fitted to identify variables associated with ubiquitous connectivity.

**Results:**

One thousand three hundred thirty-six students were included and six questionnaires (0.45%) were eliminated due to duplication of information. Fifty-four percent of participants were female, and the mean age was 16.31 ± 0.84 years. In total, 47% of students were ubiquitously connected. Associated factors to ubiquitous connectivity included better academic performance, the need to use Internet-based technologies, engaging in ludic activities on the Web and living in the state capital.

**Conclusions:**

Ubiquitous connectivity it’s a desirable condition for strengthening health promotion programs focused on young population. Strategies including digital technology tools with potential to increase adolescent engagement should be explored and evaluated. However, it is necessary to recognize that there are additional factors that may influence the success of health promotion interventions.

## Background

Ubiquitous connectivity is defined as the ability of people to interact actively or passively on the Internet, regardless of where they are. This level of connectivity is achieved by several mechanisms, although it mainly occurs via portable computers that have continuous access to mobile broadband or wireless networks (Wi-Fi) [1].

The mobile technologies and interconnectivity both pose potential for the design of new health promotion strategies [2]. With these tools it is feasible to operate Mobile Health (mHealth), which uses mobile communication technologies to promote health by supporting health care practices [2, 3]. The primary difference between them is that the latter only provided either textual or multimedia information, with the limitation that the user had to have a computing device. In contrast, the former assumes there is permanent connection between the user and his or her mobile device and that there is bidirectional communication between the device and the individual, which extends the range of opportunities to facilitate changes in lifestyles promoted by health systems around the world [4, 5].

**Fig. 1:**
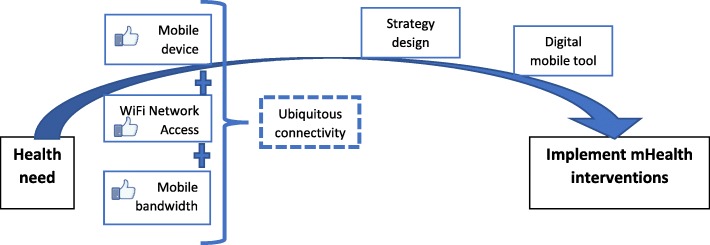
Framework linked ubiquitous connectivity and mHealth interventions

By implementing health projects in people connected in a ubiquitous way, better health results are obtained, waiting times, costs are reduced and with it a greater satisfaction in the user. With this, it is possible to manage health services differently, moving from a vertical care model (health 1.0) to an equitable and community health management model (health 2.0) [6, 7] (see Fig. 1).

Health 2.0 is a concept derived from the term “Web 2.0” that differs from “Health 1.0” (traditional view) with “Web 1.0”. In the first version, the patient is a passive entity in the continuum of care and its role is to follow given indications by the health personnel; health services are supply-oriented, i.e., the services produced are based on available resources, which do not necessarily satisfy the needs of its users, who do not participate in the design, planning or evaluation of such services. On the other hand, the concept of “Health 2.0″ recognizes that health is socially constructed, and that its diminishing or improvement runs independent of health services; rather, synergies between different actors result in healthy individuals/populations [3]. This can be achieved through the internet of things and ubiquitous connectivity because users have active participation in the management of their own health [8], from being better informed subjects to being self-managers of their health through self-care. Moreover, in an indirect manner, this can be achieved by generating information from each stage of their life that could be useful for the creation of explanatory models for collective realities and benefiting from them [3, 9].

In 2016, Mexicans users of Internet-based mobile technologies reached 65 million, with smartphones being the main one gadget (77%). Three quarters of total users are teenagers, who spend an average of 7 h and 14 min connected in the Internet [10, 11]. Their connections mainly consist of 3G or 4G networks, and social media is the preferred way of communicating with their peers [10].

In developed countries, several mHealth interventions have been designed to provide services to the adolescent population with good results, mainly in the area of sexual and reproductive health; these strategies have generated changes in behavior and received great acceptance among this age group [12–15]. However, in Mexico, which is a middle-income country and a digital adopter, there is no evidence about the digital environment in which adolescents develop, nor of the ubiquitous connectivity they have, to establish its potential as a means to health promotion interventions. Therefore, the objective of this study was to characterize the ubiquitous connectivity status of adolescents, its proportion and associated factors.

## Methods

An online survey was conducted among high school adolescents. All participants were members of a public educational subsystem which services 23 of the 33 total municipalities of the Mexican state of Morelos (located 80 km from Mexico City and with a human development index of 0.755, similar to countries such as Trinidad and Tobago and Albania.) [16]. The subsystem has two modalities: 1) Schooled, which is present in urban settings and is comprised of 12 schools; 2) Remote, which is present in 11 schools of rural communities. We only studied adolescents of the schooled modality.

A digital questionnaire was developed in Google Forms (Google LLC, v. 2016), using categories and variables similar to those evaluated in the Mexican Digital Strategy (Estrategia Digital Nacional) [17] by the Federal Institute of Telecommunications of the Government of Mexico [18], and by the Mexican Internet Association [10] and had a total of 33 items (General data -6 questions-, Individual equipment -16 questions-, School infrastructure -6 questions- and Digital habits -5 questions-). All questions used in this work are validated by the National Institute of Geography and Informatics Statistics of Mexico and were applied in the “National survey on availability and use of information technologies in households, 2017” [18].

The outcome variable, ubiquitous connectivity, was constructed by including two adolescent’s conditions: 1) having a mobile device (smart phone, tablet or both); and 2) having mobile internet access that would provide connectivity 24 h a day, 7 days a week. The independent variables were sex, having a mobile data plan, payment schemes, availability of Wi-Fi at their school, availability of a computer Center on the campus, most frequent type of activity performed on the Internet, schedules of Internet use, need to use the Internet, average grades during the last semester and school location.

### Application procedure

In each of the 12 participant schools, students in the 2nd and 4th semesters were invited to answer the digital questionnaire. All adolescents who attended the course of “Orientación educativa” (Educational Orientation) were invited to go to the Computer Center of the school, where a facilitator granted access for completion of the questionnaire within a timeframe of 30 days, time after which the responses acquisition mode was disabled. The database was revised weekly during four consecutive weeks to detect duplicate records, abnormal patterns in the answers and to determine the participation response of each of the schools. To encourage student participation, school Directors were invited to strengthen the recruitment strategy through weekly telephone reminders. The strategy used by the Directors to increase the response rate of the students was the placement of notices that reminded the students to fill out the digital survey. These notices were strategically placed in their classrooms, computer center, administrative offices, and even in the coffee shop.

### Data analysis

A descriptive analysis was performed to characterize the participants and the study variables, by using mean and standard deviation for continuous variables; absolute and relative frequencies ​​were used to describe categorical and nominal variables.

To identify associated factors to ubiquitous connectivity, a bivariate analysis was performed (Fisher’s exact test was applied for categorical variables and t-tests for continuous variables) between the outcome variable (ubiquitous connectivity) and the covariates. Finally, to identify the independent effect of each variable, those variables showing a *p*-value< 0.10 were included in a multivariate logistic regression analysis, after evaluating collinearity and interaction through backward stepwise elimination method. Hosmer-Lemeshow test was performed to measure goodness of fit. Statistical analyses were performed in Stata v.12 (StataCorp, Lakeway, TX).

## Results

Responses were received from 11 schools; one of them was eliminated because of loss of connectivity due to weather conditions. A total of 1336 students were surveyed (6 entries were eliminated due to duplicate answers), of whom 720 (53.9%) were female. The average age was 16.31 ± 0.84 years, and the average of school performance was 8.05 ± 1.04 (scale 0 to 10).

In total, 89.1% of the adolescents had a mobile device (phone and/or tablet); being smartphone the most frequent mobile device used to connect to the Internet. Android was the most commonly used operating system, and the usual version was 4.4 (KitKat). The students had been using a mobile device for an average of 13.91 ± 11.91 months, and the most commonly purchased brand was Samsung® (see Table 1).

**Table 1:** Ubiquitous connectivity and individual technological infrastructure

Ubiquitous connectivity	
- Yes	628 (47.0%)
Preferred equipment used to connect to Internet	
- Smartphone	925 (69.2%)
- Computer	305 (22.8%)
- Tablet	85 (6.4%)
- Videogame console	15 (1.1%)
- Smart television	6 (0.4%)
Operating System	
- Android	997 (78.9%)
- iOS	134 (10.6%)
- Windows	72 (5.7%)
- Other	60 (4.8%)
Mobile device age (mean)	13.91 ± 11.91 months
Data plan on the device	
- Yes	538 (42.8%)
Preferred Network	
- WiFi Home	847 (66.5%)
- Cellular Network (3G or 4G)	228 (17.9%)
- WiFi Public	133 (10.4%)
- WiFi School	47 (3.7%)
- Other	18 (1.4%)
Availability of 4G network	
- Yes	394 (31.4%)
Type of contract with telecommunications provider	
- Prepaid	713 (53.4%)
- Postpaid	495 (37.1%)
Wireless telecommunications provider	
- Telcel®	865 (64.7%)
- Movistar®	221 (16.5%)
- Unefon®	83 (6.2%)
- AT & T®	63 (4.7%)
- Other	6 (0.4%)

Regarding school infrastructure 8 out of 11 schools had Wi-Fi connectivity. However, in 85% of those schools difficulties to connect were mentioned; additionally, the web surfing speed was slow or very slow. Almost all the students had access to a computing center, although about half of the students said they used it very rarely or never (see Table 2).

**Table 2:** Digital school infrastructure of participants

Availability of Campus with WiFi	
- Yes	1062 (79.5%)
Password to connect	
- Yes	762 (61.6%)
Connection access	
- Very easy/easy	178 (14.9%)
- Difficult/very difficult	897 (85.1%)
Speed to navigate	
- Fast/very fast	177 (14.8%)
- Slow/very slow	892 (85.2%)
Availability of Campus with computer center	
- Yes	1228 (91.9%)
Use of the computer center	
- Frequent/very frequent	626 (46.8%)
- Rare/never	710 (53.2%)

About digital habits, the main activity adolescents engage in the Internet is browsing social media and looking-up information for homework. When using their mobile device, instant messaging services (WhatsApp, Facebook Messenger) were the most commonly used.

The students used their mobile device for an average of 6.38 ± 5.13 h a day, and their favorite application was WhatsApp. Almost all respondents connected at least once a week to the Internet, and half of them did so daily. The participants reported their most intense activity was during the afternoon (from 1 to 7 pm); this was observed in two-thirds of the respondents.

Almost half of the mobile device users indicated having a mobile data plan, and 2 out of 3 respondents mentioned that they most frequently accessed the Internet via Wi-Fi at the household.

About one-third of the users had 4G mobile broadband, and a single company served 64.7% of the users. The “pre-paid” modality was most commonly used (see Table 3).

**Table 3:** Digital habits of participants

Need to use Internet	
- Always	513 (44.5%)
- Weekday	412 (35.7%)
- Weekends	228 (19.8%)
- Doesn’t use it	16 (1.6%)
Time of day for most frequent use of Internet	
- Morning	61 (5.3%)
- Afternoon	814 (70.6%)
- Night	278 (24.1%)
Main activity in the internet	
- Social networks	596 (44.6%)
- Homeworks	530 (39.7%)
- Videos	128 (9.6%)
- Games	53 (4.0%)
- Others	29 (2.2%)
Time spend using the mobile devices (mean per day)	6.38 ± 5.13 h

High school performance (odds ratio [OR]: 1.37; Confidence Interval 95% [CI95%]: 1. 16-1.6); residence in the state capital (OR: 2.33, CI95%: 1.62–3.35); engaging in ludic activities such as participating in social media, streaming videos or gaming (OR 1.87, CI95%: 1.36–2.58); and having the permanent need to interact digitally (OR: 10.29, CI95%: 7.5–14.11); were factors positively associated with ubiquitous connectivity of the surveyed adolescent students (see Table 4).

**Table 4:** Associated factors with ubiquitous connectivity. Logistic regression model^a^

Variable		Ubiquitous connectivity (*n* = 998)		
		OR	95% CI	*p*-value
Score of school performance [in Mexico a numerical scale is used, the minimum approving is 6 (lowest) and 10 the highest]	Highest	5.48	4.64–6.40	< 0.0001
	Lowest	1		
Main internet activity	Homework	1		
	Entertainment	1.87	1.36–2.58	< 0.001
Need to interact digitally	Sporadically	1		
	Everyday	10.29	7.50–14.11	< 0.001
School location	South/East	1		
	Metropolitan capital area	2,34	1.62–3.35	< 0.001

^a^ Goodness of fit: 0.513

## Discussion

The main finding of this study was the identification of a significant proportion of adolescents residing in urban areas in Mexico that have a ubiquitous connectivity. This allows them to take advantage of (and be part of) the technological development of the twenty-first century. However, more than half of survey respondents lack access to wireless networks (home, school or public Wi-Fi) or do not have mobile broadband services (3G or 4G), a situation prevalent throughout the country [19].

At the end of the twentieth century, efforts to include people digitally involved equipping them with personal computers and providing them with a wire Internet connection [20]. The data obtained in this study confirm that such objective has been almost entirely fulfilled in the adolescent population (more than 98% connect weekly), and 9 out of 10 have a mobile device. However, although they are now connected by Wi-Fi, their usage scheme remains sporadic. This poses a barrier for its potential use in conducting health interventions that engage individuals permanently, and for taking advantage of the benefits generated by ubiquitous connectivity and the Internet of things [21].

Another finding to highlight is the time that adolescents allocate to use their mobile devices, which is like that of formal education. Perhaps schools, where lower amounts of time for using Internet-based technologies were reported, do not grant full connectivity to its students (i.e., they have broadband services but access to websites is restricted). This indicates that the national digital strategy must be reoriented [17].

In schools, teachers commonly prohibit the use of mobile devices, arguing that they are “distractors” [22]. This statement is valid if there are no instructional designs that take advantage of these technologies or if the school infrastructure is limited in them. However, although the connectivity offered by the schools is low in user capacity and speed, the high proportion of students that have a ubiquitous connection (through mobile broadband), opens a window of opportunity for the redesign of educational models and implementing ad hoc educational strategies that fit the technological reality of adolescents [23]. The current technological lifestyle of the young people could be used to perform and evaluate digital actions to promote health (which is a recommendation of the Pan American Health Organization) [24], which should incorporate the digital environments used by adolescents (social networks, instant messaging and games) [25, 26].

The factors associated with ubiquitous connectivity in urban adolescents found in this study break with the traditional beliefs of parents and teachers, who perceive the use of these technologies as a distractor that diminish their academic performance. Our results show that the students with better grades are those who are more connected, and although almost all students have a smartphone or tablet. Connectivity, apparently, is what makes the difference.

Coupled with connectivity, the “new digital literacy” generates the gap in academic performance between those who have ubiquitous connectivity and those who do not. On the one hand, the most vulnerable young people do not have the necessary skills to make mobile technologies empower their development, and on the contrary, they only use them as a means of entertainment [27]. On the other side, there are the adolescents who have the skills and the adequate support for personal development (and not only academic development) to be strengthened by the adoption of these technologies. However, schools that do not adapt their educational models and teaching practices to the new digital scenario prevents their students from moving forward digitally and socially.

Finally, although ubiquitous connectivity is favored because of living in large cities (which does not generate equity), the expansion of the coverage in Mexico could be improved by a project called “Shared Network”. This project uses the 700 MHz spectrum and 30,000 km of optical fiber of the public enterprise that provides the electricity services in the country (Comisión Federal de Electricidad); it is intended to bring mobile and high-speed connectivity to 92.2% of the population (including rural areas in which it would have a greater impact) in a relatively short period (5 years), with an investment of USD 7 billion. This project could position Mexico amid the countries with the most ubiquitous connectivity within the Organization for Economic Co-operation and Development (OECD) [28, 29].

## Conclusions

Ubiquitous connectivity has a great potential as a tool for strengthening health promotion programs. However, to guarantee migration in health management models middle-income Countries must also invest in fostering personnel skills to be competent in this new social reality and reconfigure the way health services are provided to the population. Decision makers should be aware of the potential value of this digital reality, taking it into account when developing new models of health promotion focused on young populations.

Strategies including digital technology tools with potential to increase adolescent engagement should be explored and evaluated. However, it is necessary to recognize that there are additional factors that may influence the success of health promotion interventions. There is no doubt that the level of online connectivity is a prerequisite for online health promotion; however, there may be some other factors that have to be taken into consideration.

Intersectoriality is crucial to perform health interventions for adolescents in educational spaces and labor. Moreover, the construction of the strategies must be participatory, as has been documented in previous reports [30–32] it is required to generate tools with permanent feedback and flexibility to achieve a sense of belonging in the users. To reach this goal, these strategies must overcome the single operation of health promotion programs to move toward the management of a healthy digital community.

## Data Availability

The data that support the findings of this study are available from National Institute of Public Health of Mexico, but restrictions apply to the availability of these data, which were used under license for the current study, and so are not publicly available. Data are however available from the authors upon reasonable request and with permission of National Institute of Public Health.

## Abbreviations

COBAEM: Colegio de Bachilleres del Estado de Morelos
OECD: Organization for Economic Co-operation and Development
OR: Odds ratio
